# Decoding cellular vulnerabilities for interpretable drug response prediction

**DOI:** 10.1093/bib/bbag559

**Published:** 2026-10-15

**Authors:** Chonghao Chen, Yulei Du, Yaxin Jiang, Yuanfang Wang, Di Tang, Xiaofan Ding, Mian He

**Affiliations:** Faculty of Health Sciences, University of Macau, Avenida da Universidade, Taipa, Macao SAR 999078, China; Faculty of Health Sciences, University of Macau, Avenida da Universidade, Taipa, Macao SAR 999078, China; Faculty of Health Sciences, University of Macau, Avenida da Universidade, Taipa, Macao SAR 999078, China; Department of General Surgery, The Seventh Affiliated Hospital of Sun Yat-sen University, No. 628 Zhenyuan Road, Guangming District, Shenzhen, Guangdong 518107, China; Department of General Surgery, The Seventh Affiliated Hospital of Sun Yat-sen University, No. 628 Zhenyuan Road, Guangming District, Shenzhen, Guangdong 518107, China; Faculty of Health Sciences, University of Macau, Avenida da Universidade, Taipa, Macao SAR 999078, China; MOE Frontiers Science Center for Precision Oncology, University of Macau, Avenida da Universidade, Taipa, Macau SAR 999078, China; Department of General Surgery, The Seventh Affiliated Hospital of Sun Yat-sen University, No. 628 Zhenyuan Road, Guangming District, Shenzhen, Guangdong 518107, China

**Keywords:** drug response prediction, deep learning, gene dependency, interpretability, precision oncology

## Abstract

Accurate prediction of anticancer drug response remains challenging because conventional approaches based on chemical structures and baseline molecular profiles do not directly capture drug-induced perturbations or cellular dependencies. Here, we present vulnerability-guided cascaded attention transformer (VCAT), which integrates Connectivity Map (CMap)/Library of Integrated Network-based Cellular Signatures (LINCS)-derived consensus transcriptional signatures with clustered regularly interspaced short palindromic repeats (CRISPR)-supervised cellular representations through adaptive encoders and dual stream cascaded attention. Across four data splitting strategies, VCAT outperformed conventional machine learning and published deep learning models, achieving the best performance in 11 of 12 classification split metric comparisons. Ablation analyses showed that CRISPR-derived vulnerability features complement baseline gene expression and that the proposed interaction architecture further improves prediction. VCAT also enhanced continuous IC50 prediction and generalized across Cancer Therapeutics Response Portal (CTRP), Profiling Relative Inhibition Simultaneously in Mixtures (PRISM), patient-derived organoids, and hepatocellular carcinoma cohorts. Attention based prioritization and integrated gradients jointly highlighted biologically relevant genes, while knockdown of cytosolic iron-sulfur assembly component 3 (CIAO3), methylenetetrahydrofolate dehydrogenase 2 (MTHFD2), and WD repeat domain 1 (WDR1) increased sorafenib sensitivity in hepatocellular carcinoma cell lines. These findings support integrating drug-induced perturbations with genetic vulnerabilities for accurate, interpretable, and transferable anticancer drug response prediction.

## Introduction

Predicting individual responses to anticancer therapies remains challenging because of intertumoral and intratumoral heterogeneity [1, 2]. The Cancer Genome Atlas (TCGA) has characterized tumor multi-omics landscapes and supported targeted therapy development [3, 4]. However, drug resistance and limited treatment benefit highlight the need for computational approaches to predict drug sensitivity and explain treatment response [5, 6].

Pharmacogenomic resources, including Cancer Cell Line Encyclopedia (CCLE) [7], Genomics of Drug Sensitivity in Cancer (GDSC) [8], CTRP [9], and PRISM [10], have enabled data-driven prediction. Existing methods combine chemical representations with molecular profiles using graph based, metric learning, and domain adaptation architectures [11–13], but their generalization and biological interpretability remain limited.

Two limitations are particularly important. First, baseline expression and mutation profiles describe cellular state but do not directly measure functional dependencies [11–14]; CRISPR screens such as DepMap provide complementary gene essentiality information [15]. Second, Simplified Molecular Input Line Entry System (SMILES) strings and molecular graphs encode chemical structure but not the downstream transcriptional consequences of drug exposure [11–13]. Drug-induced perturbation signatures may, therefore, provide a more biologically grounded representation of drug action.

We developed VCAT to integrate CMap LINCS consensus transcriptional signatures (CTSs) with CRISPR-derived cellular vulnerability representations. CTSs capture standardized drug-induced transcriptional effects, while CRISPR pretraining captures functional vulnerabilities beyond baseline expression. Adaptive encoders and dual stream cascaded attention integrate these biologically informed representations.

We compared VCAT with conventional machine learning and published deep learning models across four partitioning strategies. Representation-controlled comparisons, ablations, and redundancy analyses assessed CTS, vulnerability pretraining, and architectural contributions. We evaluated continuous IC50 prediction and external generalization and used integrated gradients (IG) and gene knockdown to assess the biological relevance of attention prioritized genes.

## Materials and methods

### Data collecting and processing

Baseline expression and CRISPR-Cas9 gene effect scores were obtained from DepMap (24Q2) [15]. GDSC served as the primary training resource, while CTRP and PRISM were reserved for external validation [8]. Cell line and compound identifiers were harmonized across datasets. CMap LINCS 2020 Level 4 profiles supplied drug-induced transcriptional features [16].

The 2738 model genes combined LINCS landmark genes with a dependency predictive gene set reported previously [17]. Compounds were matched to DrugBank [18] to annotate approval and development status.

To evaluate clinical applicability, we further collected real-world Sorafenib response data from three independent cohorts, including 19 samples from TCGA–LIHC [4] (3 responders and 13 nonresponders), 67 samples from GSE109211 [19] (21 responders and 46 nonresponders), and 379 hepatocellular carcinoma organoid samples [20] (107 responders and 272 nonresponders). These datasets were used exclusively for downstream clinical validation.

### Binary classification of drug response

GDSC IC50 values were dichotomized into sensitive and resistant labels using published procedures [12, 21, 22]. For overlapping drug cell line pairs, the regenerated labels agreed with a reference classification in >92% of cases.

Corresponding preprocessing generated binary response labels for CTRP and PRISM.

To assess information lost through binarization, we also predicted continuous GDSC log(IC50) values without an additional logarithmic transformation. We retained drug-cell line pairs shared by the fixed classification splits and continuous-response dataset, preserving the cell-line-blind and drug-blind partitions. All retained training pairs were used without undersampling. Targets in all subsets were standardized using the training set mean and standard deviation. Neural models were trained with mean squared error, selected by validation loss, and evaluated on the original log(IC50) scale using RMSE, MAE, Pearson’s and Spearman’s correlations, and *R*^2^. VCAT was compared with Random Forest, DeepCDR, PANCDR, and GCNPath under identical partitions.

### Consensus transcriptional signature generation

CMap LINCS 2020 Level 4 profiles measured 24 h after treatment were retained to control temporal variation [16, 23]. Because compounds were profiled across heterogeneous cell lines, doses, and replicate numbers, we derived a CTS for each drug at a 10 μM reference dose. One of four estimators was selected for each drug-gene pair according to data availability.

#### Linear mixed-effects model for high-density observations

For drug–gene pairs with sufficient data density, defined as at least four observations spanning at least two cell lines and at least two distinct dose levels, we fitted a linear mixed-effects model to capture both dose dependence and cell line heterogeneity:


$$ {Z}_{ijk}={\beta}_0+{\beta}_1{\log}_{10}\left({d}_k\right)+{u}_j+{\epsilon}_{ijk} $$

where ${Z}_{ijk}$ denotes the level 4 Z-score for gene *i* under a given compound in cell line *j* at dose ${d}_k$, ${\beta}_0$ is the intercept, ${\beta}_1$ is the dose response slope, ${u}_j\sim N\left(0,{\tau}^2\right)$ represents the random effect for cell line (*τ* is the standard deviation), and ${\epsilon}_{ijk}$ is the residual error. The consensus Z-score at the reference dose ${D}_{\mathrm{ref}}=10\ \mathrm{\mu} \mathrm{M}$ was estimated as


$$ {\overset{\wedge }{Z}}_{\mathrm{ref}}={\overset{\wedge }{\beta}}_0+{\overset{\wedge }{\beta}}_1{\log}_{10}\left({D}_{\mathrm{ref}}\right) $$

#### Intercept only mixed models for single-dose, multi context-data

For multi cell line observations available at one dose, an intercept-only mixed model was fitted and attenuated according to the distance from the reference dose:


$$ {\overset{\wedge }{Z}}_{\mathrm{ref}}={\overset{\wedge }{\beta}}_0\cdotp \lambda, \mathrm{where}\ \lambda =\frac{1}{1+\alpha \left|{\log}_{10}\left({d}_{\mathrm{obs}}\right)-{\log}_{10}\left({D}_{\mathrm{ref}}\right)\right|} $$

where *α* is a scaling parameter controlling the degree of attenuation as the observed dose deviates from the reference dose.

#### Gaussian weighted aggregation for sparse data

For drug–gene pairs with only two or three observations, where mixed effects models were unstable or failed to converge, we estimated the consensus signal using a Gaussian-weighted average. Each observation was weighted according to the proximity of its dose to the reference concentration:


$$ {\overset{\wedge }{Z}}_{\mathrm{ref}}=\frac{\sum_{\kappa }{w}_k{Z}_k}{\sum_{\kappa }{w}_k},{w}_k=\exp \left(-\frac{{\left({\log}_{10}\left({d}_k\right)-{\log}_{10}\left({D}_{\mathrm{ref}}\right)\right)}^2}{2{\sigma}^2}\right) $$

where ${\sigma}^2$ denotes the bandwidth controlling the smoothness of the Gaussian weighting. This approach prioritizes observations closer to $10\ \mathrm{\mu} \mathrm{M}$ while incorporating all available data.

#### Single point extrapolation for minimal observations

For drug–gene pairs with only a single available observation, the consensus Z-score was estimated by direct extrapolation with a conservative distance penalty:


$$ {\overset{\wedge }{Z}}_{\mathrm{ref}}={Z}_{\mathrm{obs}}\cdotp \frac{1}{1+\left|{\log}_{10}\left({d}_{\mathrm{obs}}\right)-{\log}_{10}\left({D}_{\mathrm{ref}}\right)\right|} $$

These estimators produced standardized CTSs despite variable LINCS coverage.

The CTS matrix served two purposes. Figure 2A–E analyze the standardized CTS input by Uniform Manifold Approximation and Projection (UMAP), clustering, and pathway, mechanism-of-action, and target-family enrichment to assess its biological structure. During VCAT training, the adaptive drug encoder transforms each CTS vector into a task-specific embedding optimized jointly with the cellular representation. Thus, Fig. 2 evaluates the input representation rather than the learned embedding.

### Architecture of the VCAT framework

VCAT contains a CRISPR-pretrained cell encoder, an adaptive CTS encoder, and a dual stream cascaded interaction module (Fig. 1A).

**Figure 1:**
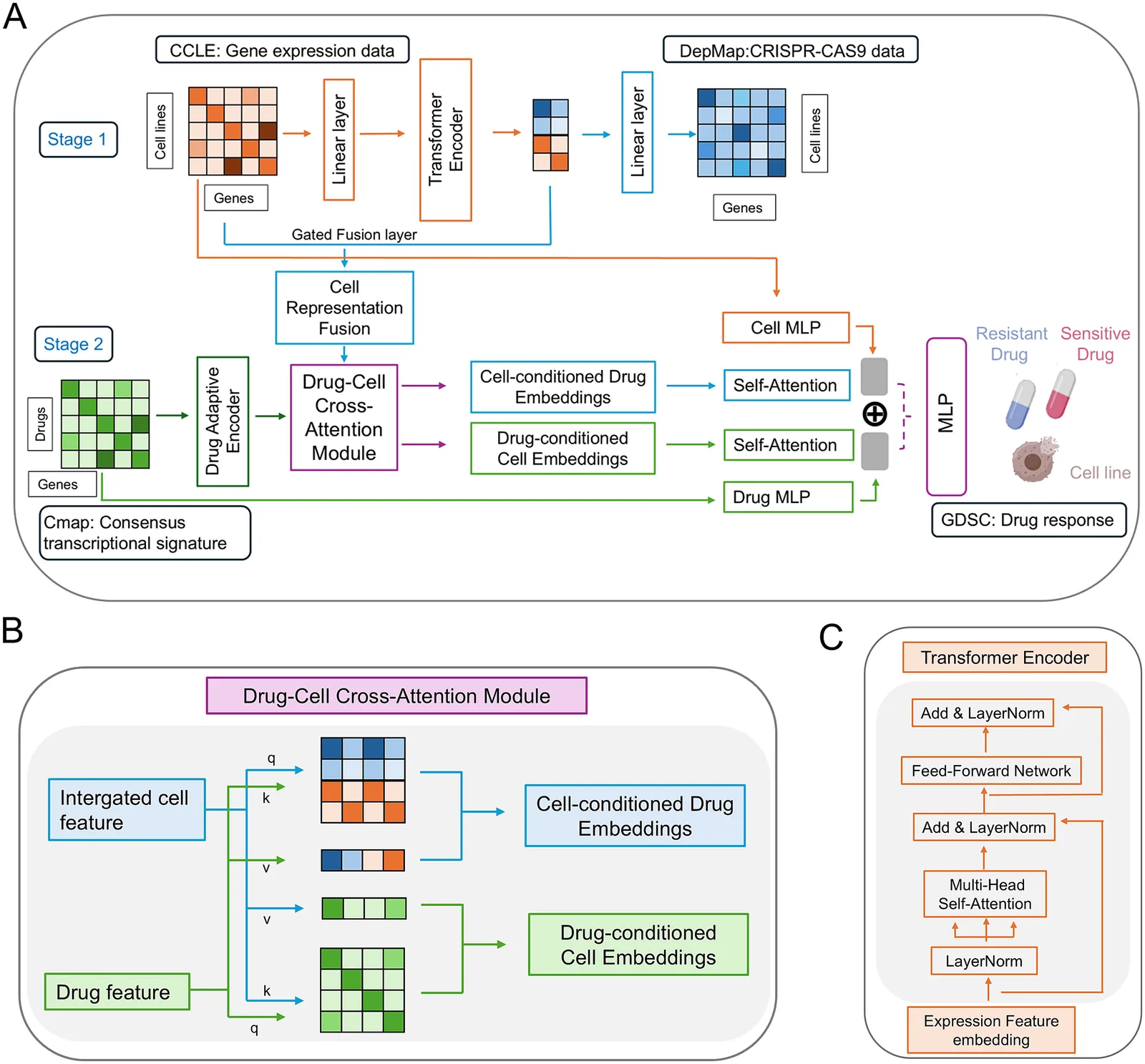
Schematic illustration of the vulnerability-guided cascaded attention transformer for drug response prediction. (A) Overview of deep learning model VCAT. (B) Structure of the drug-cell cross-attention module, in which integrated cell features and drug features are used to generate cell-conditioned drug embeddings and drug-conditioned cell embeddings. (C) Structure of the transformer encoder used in the vulnerability-aware cell representation module.

#### Biologically informed cell encoder

For each cell line, the standardized CCLE expression profile ${X}_i\in{\mathrm{R}}^G$ was converted into two gene-level representations. The expression representation ${H}_{\mathrm{expr}}\in{\mathrm{R}}^G$ was generated by projecting each scalar gene-expression value into a *d*-dimensional token using a learnable linear layer.

The vulnerability prediction module (VPM) projected expression into gene tokens, added gene-position embeddings, and processed them with a Transformer encoder. A shared head predicted DepMap CRISPR-Cas9 gene effect scores using a combined mean-squared-error and Pearson correlation loss.

The hidden gene-level states immediately before the dependency-prediction head were retained as ${H}_{\mathrm{vpm}}$. Importantly, CRISPR dependency scores were used only as pretraining targets and were not required as model inputs during downstream training or inference. Thus, ${H}_{\mathrm{vpm}}$ represents a CRISPR-supervised transformation of baseline expression rather than a direct CRISPR measurement.

The two representations were projected into a common latent space and combined using a learnable gate:


$$ {G}_i=\sigma \left(\mathrm{MLP}\left(\left[{H}_{\mathrm{expr}};{H}_{\mathrm{vpm}}\right]\right)\right),{H}_{\mathrm{cell}}={G}_i\odot{H}_{\mathrm{expr}}+\left(1-{G}_i\right)\odot{H}_{\mathrm{vpm}} $$

where σ denotes the sigmoid function, $\left[\cdotp; \cdotp \right]$ denotes concatenation, and *⊙* denotes element-wise multiplication. A self-attention block was subsequently applied to the fused gene tokens to generate the final cellular representation.

#### Adaptive context gated drug encoder

The drug encoder converted each CTS vector into a task specific representation through parallel local and global branches. The local branch applied a gated multilayer perceptron (MLP) to individual perturbation scores, while the global branch used the full CTS profile to modulate gene level features.

A feature analyzer processed the complete CTS vector and generated two drug specific fusion weights through a softmax function. The final drug representation was calculated as:


$$ {H}_{\mathrm{drug}}={\alpha}_{\mathrm{local}}{H}_{\mathrm{local}}+{\alpha}_{\mathrm{global}}{H}_{\mathrm{global}} $$

where ${\alpha}_{\mathrm{local}}+{\alpha}_{\mathrm{global}}=1$. This adaptive fusion enables the encoder to balance individual gene perturbations and global transcriptional context for each drug. The resulting ${H}_{\mathrm{drug}}$ is a learned embedding optimized for response prediction and is distinct from the CTS input analyzed in Fig. 2.

**Figure 2:**
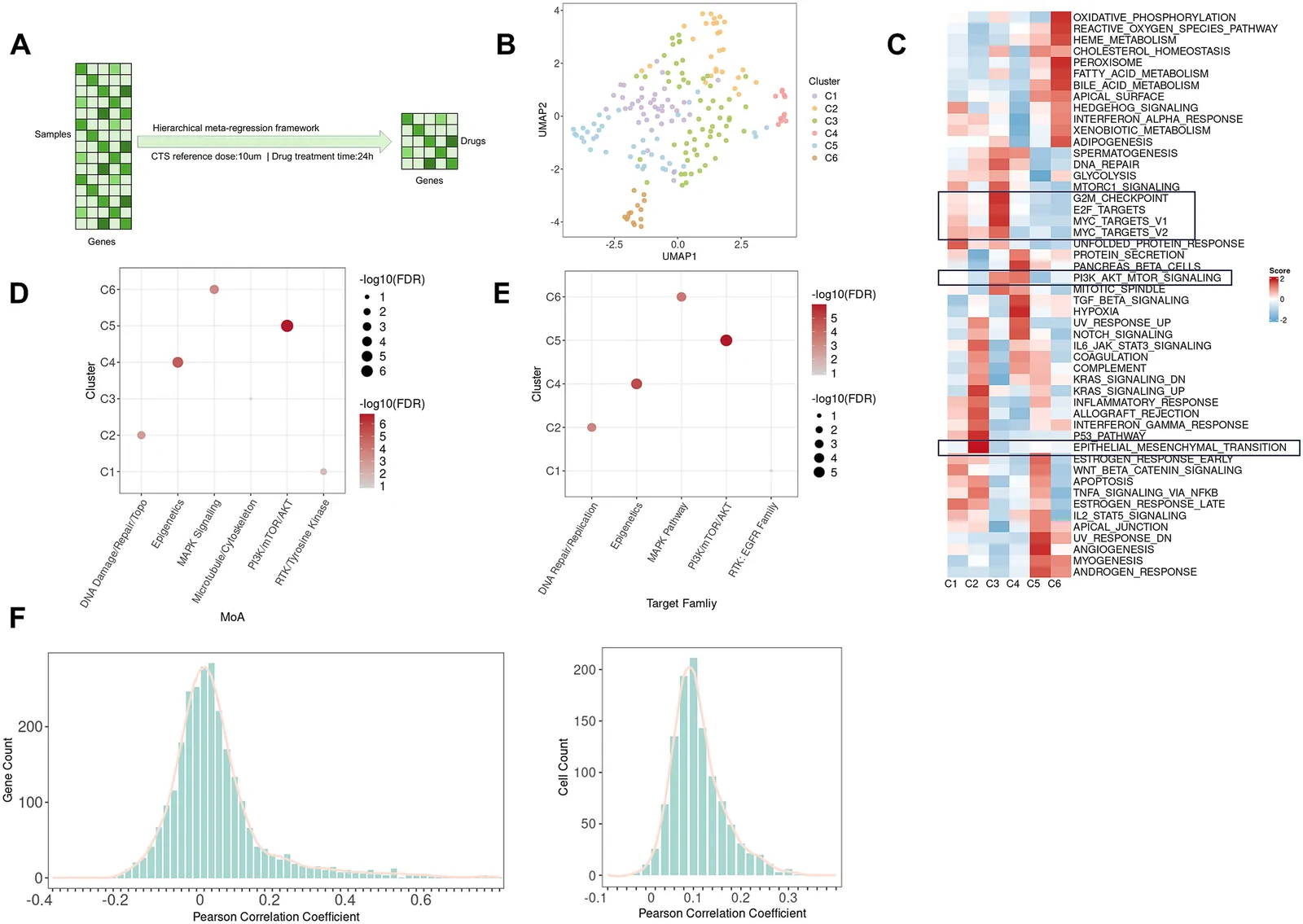
Construction and biological characterization of consensus transcriptional signatures and evaluation of the vulnerability-informed cell encoder. (A) Schematic overview of the hierarchical meta-regression framework used to derive CTSs from CMap perturbation profiles under heterogeneous experimental conditions, standardized to a reference dose of 10 um and a treatment time of 24 h. (B) UMAP visualization of the standardized CTS input profiles, showing six distinct transcriptional perturbation clusters (C1–C6). (C) Hallmark pathway enrichment heatmap for the six CTS clusters. (D) Fisher’s exact test for the association between CTS-defined clusters and drug MoA. (E) Fisher’s exact test for the association between CTS-defined clusters and drug target families. (F) Distribution of Pearson’s correlation coefficients between predicted dependency scores and DepMap true dependency scores at the gene level (left) and cell line level (right).

#### Dual stream cascaded interaction module

At each cascaded interaction layer, cell features first queried drug features:


$$ {H}_{c-d}^{(l)}=\mathrm{CrossAttn}\left(Q={H}_{\mathrm{cell}}^{\left(l-1\right)},K={H}_{\mathrm{drug}}^{\left(l-1\right)},V={H}_{\mathrm{drug}}^{\left(l-1\right)}\right) $$

Second, drug features queried cell features to identify context-specific vulnerabilities and response-related pathways:


$$ {H}_{d-c}^{(l)}=\mathrm{CrossAttn}\left(Q={H}_{\mathrm{drug}}^{\left(l-1\right)},K={H}_{\mathrm{cell}}^{\left(l-1\right)},V={H}_{\mathrm{cell}}^{\left(l-1\right)}\right) $$

Thirdly, both streams are updated via self-attention to consolidate the interaction information.

A parallel bidirectional attention branch pooled cell and drug tokens with learnable queries and supplied complementary interaction features to the prediction head.

#### Global shortcut and prediction head

To preserve coarse grained information that might be attenuated during deep interaction modeling, we introduced a global shortcut branch. In this branch, the raw cell and drug input vectors were processed independently by MLPs to generate global representations ${V}_{\mathrm{global}}^{\mathrm{cell}}$ and ${V}_{\mathrm{global}}^{\mathrm{drug}}$. The final predictive representation was obtained by concatenating pooled interaction features and global shortcut features:


$$ {V}_{\mathrm{final}}=\!\left[{\mathrm{V}}_{\mathrm{pool}}^{\mathrm{cell}}\left\Vert{\mathrm{V}}_{\mathrm{pool}}^{\mathrm{drug}}\right\Vert \left({V}_{\mathrm{pool}}^{\mathrm{cell}}\circ{V}_{\mathrm{pool}}^{\mathrm{drug}}\right)|\Big\Vert{V}_{\mathrm{pool}}^{\mathrm{cell}}-{\mathrm{V}}_{\mathrm{pool}}^{\mathrm{drug}}|\left\Vert{\mathrm{V}}_{\mathrm{global}}^{\mathrm{cell}}\right\Vert{V}_{\mathrm{global}}^{\mathrm{drug}}\right] $$

where *∘* denotes element-wise multiplication. The concatenated vector was then fed into an MLP classifier to predict the probability of drug sensitivity.

#### Loss function and model training

VCAT was trained for binary response using binary cross-entropy with label smoothing:


$$ {y}_{\mathrm{smooth}}={y}_{\mathrm{true}}\cdotp \left(1-\epsilon \right)+\left(1-{y}_{\mathrm{true}}\right)\cdotp \epsilon $$

The final training loss was defined as


$$ L=-\frac{1}{N}\mathop{\sum}\limits_{i=1}^N\left[{y}_{\mathrm{smooth}}^{(i)}\log \left(\sigma \left({z}_i\right)\right)+\left(1-{y}_{\mathrm{smooth}}^{(i)}\right)\log \left(1-\sigma \left({z}_i\right)\right)\right] $$

where ${z}_i$ denotes the raw model logit, $\sigma \left(\cdotp \right)$ is the sigmoid activation, and *N* is the batch size. Model parameters were optimized using AdamW with weight decay, and a ReduceLROnPlateau scheduler was applied based on validation area under the receiver operating characteristic curve (AUROC) to promote stable convergence and improve generalization.

### Data curation and experimental configuration

After harmonization, matched expression, dependency, CTS, and GDSC response data yielded 531 cell lines and 186 drugs for modeling.

Four splits were used (~80/10/10% training/validation/test). Mixed: drug-cell line pairs were randomly partitioned. Cell line-blind: validation and test cell lines were absent from training. Drug blind: validated and tested drugs were absent from training. Disjoint: neither validation/test cell lines nor drugs occurred in training.

The drug-blind setting used random partitioning at the compound identity level rather than scaffold-based splitting. This design was selected because the primary drug representation used by VCAT is a LINCS-derived transcriptional perturbation signature rather than a chemical structure. The drug-blind experiment, therefore, evaluates generalization to unseen compounds based on their biological perturbation profiles. To quantify structural overlap across these partitions, molecular structures were standardized to their fragment-parent forms and encoded using nonchiral ECFP4 fingerprints (radius = 2; 2048 bits). Across the 10 drug-identity splits, we calculated pairwise Tanimoto similarities between the training, validation, and test sets, as well as the maximum similarity of each held-out compound to the corresponding training set. The binary-response dataset exhibited an ~1:6 sensitive to resistant class ratio. Random undersampling was performed independently within the training, validation, and test sets after data partitioning to produce balanced subsets. Sampling was conducted separately within each subset, and no observations were transferred between subsets. Consequently, the reported area under the precision–recall curve (AUPRC) values correspond to evaluation sets with a balanced class distribution.

Primary classification benchmarks used ten seed-specific partitions and reported mean ± SD for AUROC, AUPRC, and F1-score. Component ablations used ten random seeds. Seed-level matched comparisons are shown in Supplementary Fig. S3. Benchmarks included five machine learning and five published deep learning models (Table 1). Representation-controlled tests added CTS to published models, replaced CTS with SMILES in VCAT, or replaced cascaded attention with concatenation and an MLP (Supplementary Table S1). Component-wise ablation experiments were performed under the cell line-blind and drug-blind settings using ten random seeds. The evaluated variants included expression-only and VPM-only cellular representations, removal of VPM pretraining, fixed expression-VPM fusion, local only and global-context-only drug encoders, fixed drug fusion, removal of cascaded attention, removal of the cell-to-drug attention branch, replacement of attention with concatenation followed by an MLP, and removal of the global shortcut branches.

For external validation, VCAT was compared with Random Forest, DeepCDR, PANCDR, and GCNPath using CTRP, PRISM, GSE109211, TCGA-LIHC, and patient derived liver cancer organoid datasets. Drug-cell line pairs overlapping with GDSC were removed from CTRP and PRISM before evaluation.

### Transcriptomic alignment via contrastive PCA

To reduce preclinical to patient domain variation, linear contrastive principal component analysis (cPCA) was applied to shared gene expression from liver cancer cell lines and each patient-derived dataset. This follows a translational dependency mapping study aligning DepMap and TCGA transcriptomes [17]. cPCA was chosen for its transparency and low overfitting risk in small cohorts relative to nonlinear adaptation [24]. It was used to remove dominant domain-specific components, not all biological differences.

For each external hepatocellular carcinoma (HCC) dataset, cPCA was fitted without using drug response labels. The first four contrastive principal components, which captured the strongest domain-specific differences, were removed from the expression profiles. The resulting aligned transcriptomes were subsequently used as cellular inputs for VCAT prediction. UMAP was used only to visualize the distributions of cell-line and patient derived samples before and after alignment and did not contribute directly to model training or prediction.

To characterize the biological information represented by the removed components, genes were ranked according to the absolute magnitude of their loadings within each of the first four contrastive principal components. Gene set enrichment analysis was performed using the 100 genes with the highest absolute loadings from each component. Enriched processes were evaluated using normalized enrichment scores and Benjamini–Hochberg-adjusted false discovery rates. The alignment distributions and enrichment results are presented in Supplementary Fig. S5.

### Model interpretation and representation analysis

To examine the information captured by the cellular representations, we extracted the expression, vulnerability, fused, and final cellular embeddings from the trained VCAT model for the same set of cell lines. Pairwise representational similarity was quantified using linear centered kernel alignment (CKA). The directional predictability between the expression and vulnerability representations was evaluated using five-fold cross-validated multi-output ridge regression, with the coefficient of determination (*R*^2^) used to quantify the variance in one representation explained by the other. Canonical correlation analysis was additionally performed after principal component reduction, retaining components explaining 90% of the variance with a maximum of 20 components. The first 10 canonical correlations were reported.

For attention-based interpretation, cross-attention weights were extracted from the cascaded interaction layers and averaged across attention heads. In the representative JHH-1 analysis, layer-specific drug-to-cell attention patterns were compared between a predicted sensitive compound and a predicted resistant compound. Genes preferentially emphasized under the resistant condition were subsequently subjected to Gene Ontology and Kyoto Encyclopedia of Genes and Genomes (KEGG) pathway enrichment analyses. These attention patterns were interpreted as hypothesis-generating associations rather than definitive causal explanations.

For the sorafenib analysis, gene-level attention scores were aggregated across HCC cell lines classified as sorafenib resistant by VCAT. The resulting average attention scores were used to rank candidate resistance-associated genes. To obtain an attribution measure independent of attention weights, we additionally applied IG to the same resistant cell line sorafenib pairs.

IG targeted the resistance score (negative sensitivity logit) using a zero baseline in training standardized expression space and 64-step trapezoidal integration. Positive signed scores indicate contributions toward resistance. Scores were averaged across resistant cell lines and compared with attention rankings.

### siRNA knockdown and drug sensitivity assay

Sorafenib was dissolved in dimethyl sulfoxide to prepare a 20 mM stock solution, which was stored at −20°C in the dark. Before use, the stock solution was diluted with the corresponding culture medium to the indicated working concentrations. Human hepatocellular carcinoma cell lines SNU387 and SNU449 were kindly provided by the laboratories of Prof. He and Prof. Deng, respectively. Cells were cultured in RPMI-1640 medium supplemented with 10% fetal bovine serum and 1% penicillin–streptomycin at 37°C in a humidified incubator with 5% CO_2_.

For gene-silencing experiments, cells in the logarithmic growth phase were seeded into six-well plates and transfected with a negative control siRNA (siNC) or siRNAs targeting CIAO3 (siCIAO3), MTHFD2 (siMTHFD2), or WDR1 (siWDR1). The siRNA sequences, written in the 5′-3′ direction, were as follows: siNC, sense UUCUCCGAACGUGUCACGU and antisense ACGUGACACGUUCGGAGAA; siCIAO3, sense GCAGGUCGCUUGCUGCAUA and antisense UAUGCAGCAAGCGACCUGC; siMTHFD2, sense GUGUGUGGGAAAUAAUCAA and antisense UUGAUUAUUUCCCACACAC; and siWDR1, sense CAUCAAGGGUCACAGUAAA and antisense UUUACUGUGACCCUUGAUG. After 24 h of transfection, cells were harvested and resuspended at a density of 1 × 10^5^ cells/ml. Subsequently, 100 μl of the cell suspension was seeded into each well of a 96-well plate and incubated for an additional 24 h before drug treatment.

Cells received sorafenib at 0.1–100 μM (seven concentrations; three technical replicates). After 72 h, viability was measured by CCK-8 after 2 h at 37°C using absorbance at 450 nm. Four-parameter logistic curves of log₁₀ concentration versus relative viability yielded IC50 values in GraphPad Prism. Three independent experiments are reported as mean ± SEM.

### Statistical analysis

Pearson correlation assessed VPM-predicted versus observed dependency scores. Fisher’s exact test evaluated MoA and target-family enrichment across CTS clusters. Hallmark, Gene Ontology, and KEGG enrichment used overrepresentation analysis [25]. Seed-matched VCAT-baseline comparisons used two-sided paired Wilcoxon signed-rank tests, with Benjamini–Hochberg adjustment within each split metric panel. Adjusted *P* values < 0.05 were considered significant. For the ablation analysis, each variant was compared with full VCAT using a two-sided paired Wilcoxon’s signed-rank test on the 10 matched seeds, with Benjamini–Hochberg correction for multiple comparisons. Adjusted *P* values < 0.05 were considered statistically significant.

**Table 1:** Performance comparison of CTS-based VCAT with conventional machine learning and SMILES-based deep learning models across four data splitting settings.

Models	Mixed			Cell line-blind			Drug-blind			Disjoint		
	AUROC	AUPRC	F1-score	AUROC	AUPRC	F1-score	AUROC	AUPRC	F1-score	AUROC	AUPRC	F1-score
Lasso	0.775 (0.008)	0.759 (0.007)	0.731 (0.008)	0.720 (0.022)	0.706 (0.039)	0.692 (0.0048)	0.722 (0.032)	0.708 (0.034)	0.695 (0.018)	0.673 (0.037)	0.670 (0.040)	0.675 (0.020)
SVM	0.773 (0.008)	0.753 (0.008)	0.730 (0.009)	0.727 (0.021)	0.714 (0.037)	0.690 (0.011)	0.731 (0.027)	0.714 (0.033)	0.703 (0.013)	0.671 (0.031)	0.665 (0.037)	0.676 (0.020)
Ridge	0.774 (0.008)	0.757 (0.007)	0.730 (0.009)	0.722 (0.021)	0.710 (0.038)	0.693 (0.009)	0.731 (0.026)	0.717 (0.032)	0.706 (0.015)	0.670 (0.027)	0.671 (0.034)	0.667 (0.029)
Elastic Net	0.772 (0.008)	0.758 (0.008)	0.731 (0.009)	0.723 (0.020)	0.710 (0.035)	0.693 (0.005)	0.732 (0.026)	0.718 (0.032)	0.699 (0.017)	0.672 (0.031)	0.672 (0.041)	0.675 (0.011)
Random Forest	0.781 (0.007)	0.783 (0.007)	0.746 (0.006)	0.752 (0.012)	**0.755 (0.019)**	0.702 (0.006)	0.731 (0.022)	0.730 (0.025)	0.671 (0.012)	0.702 (0.036)	0.705 (0.038)	0.667 (0.015)
DeepCDR Qiao *et al*. (2020) [11]	0.796 (0.015)	0.789 (0.016)	0.723 (0.015)	0.747 (0.022)	0.736 (0.023)	0.682 (0.035)	0.741 (0.019)	0.731 (0.027)	0.670 (0.024)	0.699 (0.026)	0.696 (0.035)	0.642 (0.023)
Super.FELT Sejin *et al*. (2021) [12]	0.757 (0.010)	0.738 (0.012)	0.681 (0.022)	0.717 (0.032)	0.708 (0.031)	0.639 (0.062)	0.734 (0.032)	0.733 (0.036)	0.662 (0.039)	0.693 (0.030)	0.703 (0.034)	0.623 (0.039)
PANCDR Juyeon *et al*. (2024) [13]	0.789 (0.014)	0.779 (0.013)	0.703 (0.018)	0.749 (0.015)	0.736 (0.022)	0.665 (0.032)	0.744 (0.029)	0.742 (0.033)	0.678 (0.029)	0.696 (0.040)	0.701 (0.047)	0.612 (0.058)
DRExplainer Shi *et al*. (2025) [26]	0.797 (0.009)	0.783 (0.010)	0.725 (0.009)	0.715 (0.044)	0.707 (0.034)	0.637 (0.053)	0.695 (0.035)	0.689 (0.029)	0.621 (0.047)	0.683 (0.033)	0.696 (0.038)	0.610 (0.042)
GCNPath Yoon *et al*. (2026) [27]	0.791 (0.005)	0.782 (0.007)	0.717 (0.006)	0.736 (0.026)	0.734 (0.028)	0.666 (0.035)	0.746 (0.019)	0.740 (0.025)	0.673 (0.024)	0.693 (0.035)	0.704 (0.041)	0.614 (0.051)
VCAT (Ours)	**0.809 (0.012)**	**0.800 (0.010)**	**0.750 (0.011)**	**0.765 (0.019)**	0.753 (0.025)	**0.720 (0.016)**	**0.768 (0.024)**	**0.761 (0.028)**	**0.713 (0.011)**	**0.723 (0.037)**	**0.721 (0.053)**	**0.687 (0.020)**

Values are reported as mean ± SD across 10 random seeds. Bold values indicate the best result under each split. Seed-level distributions and statistical comparisons with selected baselines are presented in Supplementary Fig. S3.

## Results

### VCAT outperforms existing models across multiple data splitting settings

VCAT was compared with five machine learning and five published deep learning baselines across mixed, cell line-blind, drug-blind, and disjoint splits. Across 10 runs, VCAT achieved the highest AUROC and F1-score in all four splits and the highest AUPRC in three, ranking first in 11 of 12 split metric comparisons (Table 1). The exception was cell line-blind AUPRC, where VCAT (0.753) was comparable to Random Forest (0.755). Structural overlap across the 10 random drug identity splits was limited overall, with a mean maximum test-to-training Tanimoto similarity of 0.350 ± 0.054 and a mean Bemis–Murcko scaffold overlap of 11.6% (Supplementary Fig. S7, Supplementary Table S3A and B).

Seed-level distributions and two-sided paired Wilcoxon’s tests against Random Forest, DeepCDR, and GCNPath are shown in Supplementary Fig. S3. After Benjamini–Hochberg correction, VCAT improved significantly over at least one comparator in all four settings for AUROC and F1-score and in mixed/disjoint AUPRC, whereas cell-line- and drug-blind AUPRC differences were nonsignificant.

Representation controlled comparisons showed that adding CTS generally improved published models, while replacing CTS with SMILES reduced VCAT AUROC from 0.768 to 0.721 in drug-blind and from 0.723 to 0.705 in disjoint evaluation (Supplementary Table S1). Thus, both CTS and its integration contribute to performance.

Continuous IC50 regression produced the best overall performance for VCAT in both cell line- and drug-blind settings (Supplementary Table S2). Gains over DeepCDR were modest for unseen cell lines but larger for unseen drugs, where VCAT reduced RMSE from 2.193 to 1.814 and increased Spearman’s correlation from 0.499 to 0.673. Performance, therefore, did not depend solely on binarized labels.

### Consensus transcriptional signatures capture distinct drug-induced phenotypic states

We analyzed the standardized CTS matrix before VCAT encoding to test whether the drug input retained biological structure. UMAP and unsupervised clustering identified six transcriptional states spanning inflammatory/stress, proliferative, developmental/immune, and metabolic programs (Fig. 2A–C). These clusters differed in pathways including interleukin-6 (IL6)–Janus kinase (JAK)–signal transducer and activator of transcription 3 (STAT3), cell-cycle, phosphoinositide 3-kinase (PI3K)/protein kinase B (AKT)/mechanistic target of rapamycin (mTOR) signaling pathway, and oxidative phosphorylation.

MoA and target family distributions were nonrandom across CTS clusters (Fisher’s exact test; Fig. 2D and E). For example, cluster 5 was enriched for PI3K/mTOR/AKT inhibitors, consistent with suppressed PI3K–AKT–mTOR signaling, whereas cluster 4 was enriched for epigenetic regulators, including HDAC inhibitors. These associations link downstream CTS phenotypes to established pharmacological mechanisms.

Figure 2A–E, therefore, establish, the biological relevance of the CTS input. During training, the adaptive encoder converts CTS into a task-specific embedding optimized jointly with cellular features; Fig. 2 does not analyze this learned embedding.

### Biologically informed representations and model components jointly improve prediction

VPM-predicted and observed dependency correlations were heterogeneous: most genes and cell lines showed weak to moderate correlations, with stronger positive correlations restricted to a subset (Fig. 2F).

To determine whether the vulnerability representation simply reproduced baseline expression, we performed representation-redundancy analyses (Supplementary Fig. S4). Linear CKA between the expression and vulnerability representations was 0.42, indicating moderate global similarity. Cross-validated prediction showed that expression explained ~77% of the variance in the vulnerability representation, whereas the vulnerability representation explained only ~29% of expression variance. Canonical correlations also progressively decreased across successive components. These findings indicate that the two representations share several dominant axes but retain substantial nonredundant information. The vulnerability representation therefore constitutes a CRISPR-supervised reorganization of baseline expression rather than an independent CRISPR measurement during inference.

We next performed a systematic component-wise ablation analysis across ten random seeds under the cell line-blind and drug-blind settings (Fig. 3). Full VCAT achieved AUROC/AUPRC values of 0.767/0.762 and 0.763/0.755, respectively, significantly outperforming all evaluated ablated variants in both metrics and settings. Expression-only and VPM-only variants both showed reduced performance, supporting the complementary contribution of the two cellular representations. Removing VPM pretraining or replacing adaptive expression-VPM fusion with fixed fusion also decreased performance. Similarly, using only the local or global drug context, or replacing their adaptive integration with fixed fusion, resulted in lower predictive performance. Ablating cascaded or cell-to-drug attention, replacing attention with concatenation and an MLP, or removing global shortcuts also reduced performance. The largest drop followed shortcut removal (0.708/0.701 cell line-blind; 0.696/0.692 drug-blind), supporting complementary contributions from these components.

**Figure 3:**
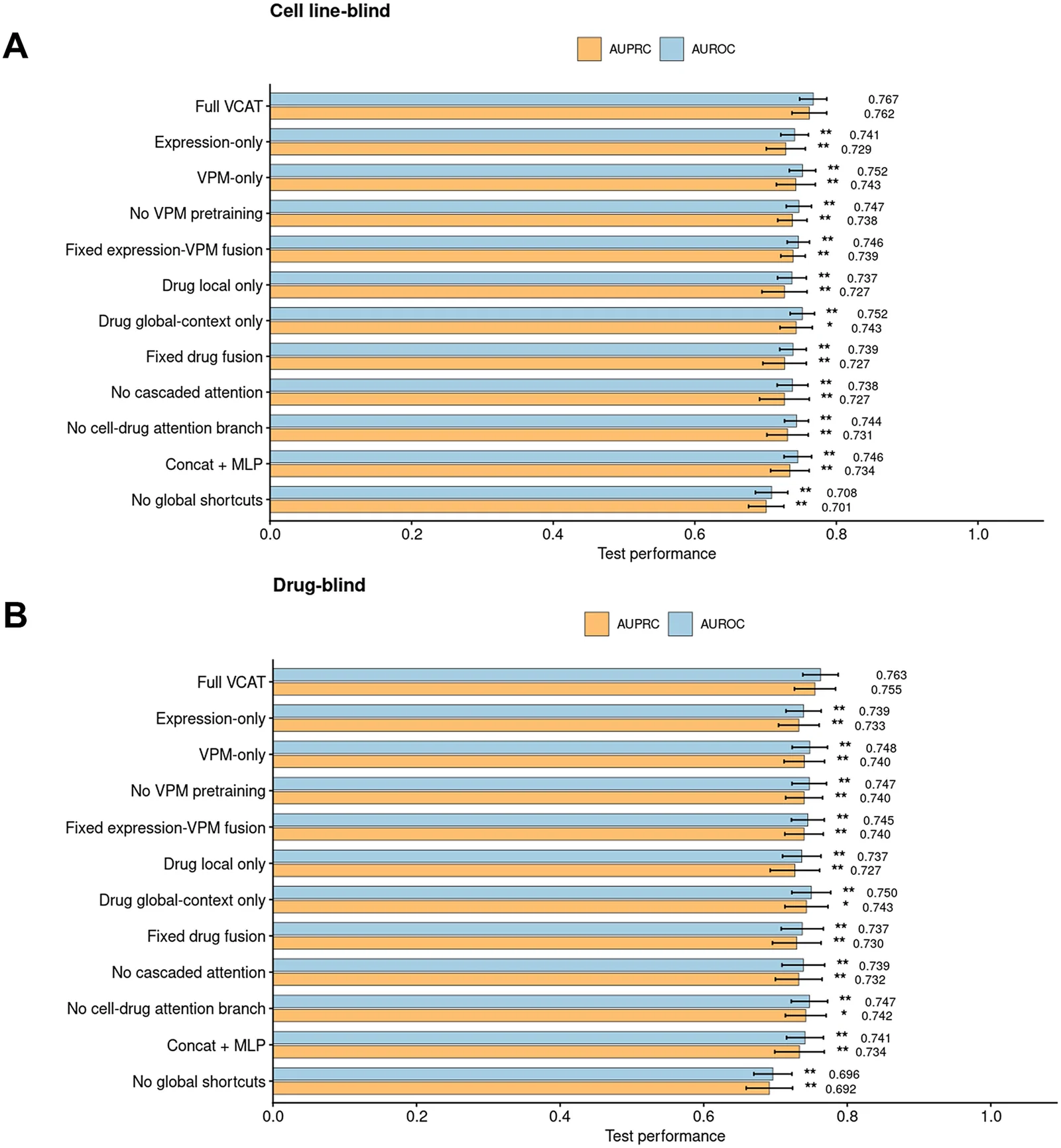
Component-wise ablation analysis of VCAT**.** Performance of the complete VCAT model and its ablated variants under the (A) cell line-blind and (B) drug-blind settings. The evaluated variants assess the cell line encoder, drug encoder, cascaded interaction module, and global shortcut branch. Results are reported as mean ± SD across 10 random seeds. Each ablated variant was compared with full VCAT using a two-sided paired Wilcoxon’s signed-rank test, with Benjamini–Hochberg correction for multiple comparisons. *, **, and *** denote adjusted *P* < 0.05, *P* < 0.01, and *P* < 0.001, respectively.

### Cascaded attention reveals distinct molecular programs associated with resistance

Attention patterns were examined in JHH-1 for PD-184352 (predicted sensitive, 0.78) and YK-4279 (predicted resistant, 0.11; Fig. 4A). Drug-to-cell attention was compared across interaction layers.

**Figure 4:**
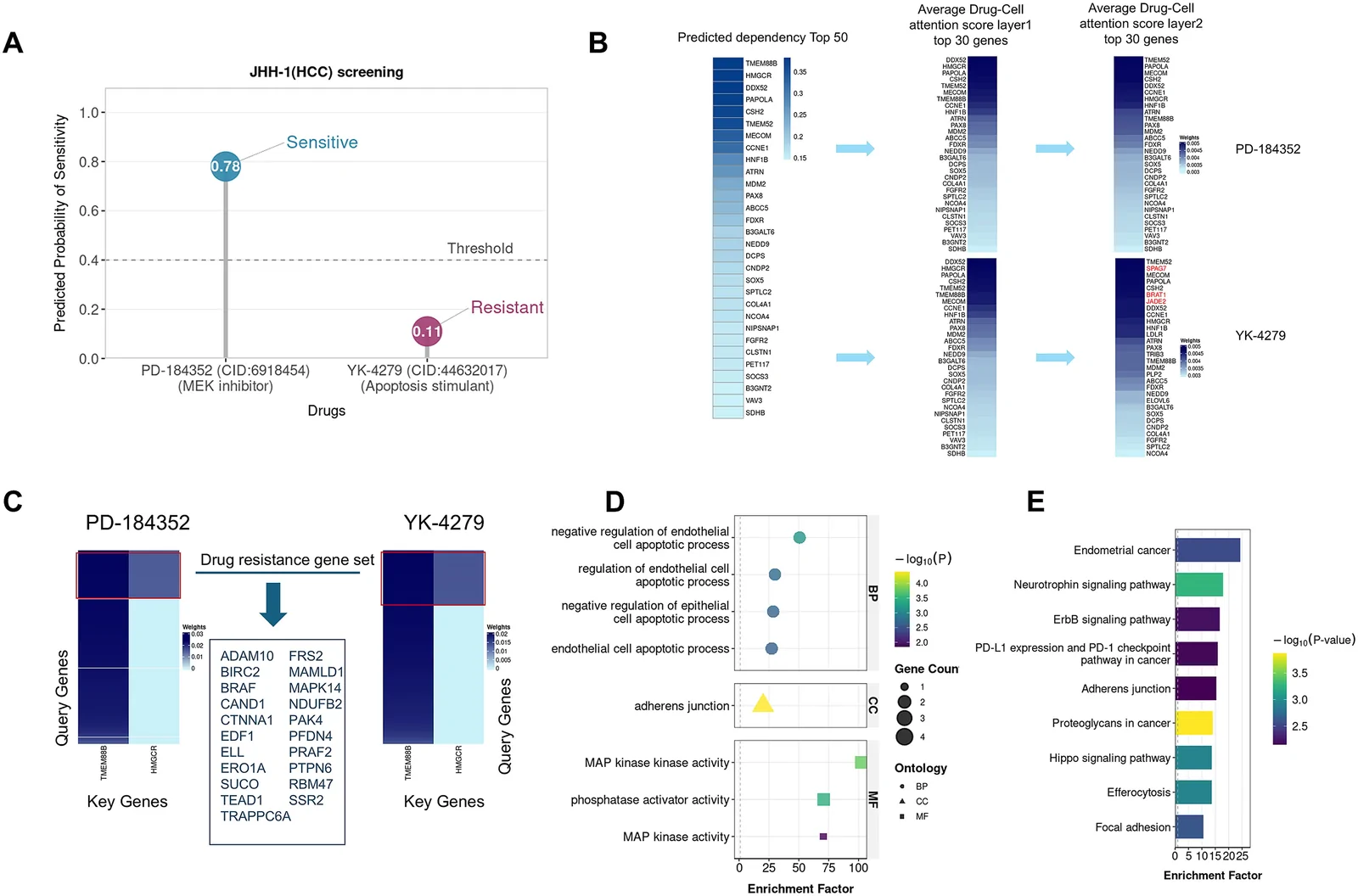
Interpretable analysis of drug sensitivity and resistance using the cascaded attention module. (A) Predicted sensitivity probabilities for two representative compounds in the JHH-1 hepatocellular carcinoma cell line: PD-184352, predicted as sensitive, and YK-4279, predicted as resistant. The dashed line indicates the classification threshold. (B) Comparison of attention patterns across cascaded interaction layers. The left panel shows the top predicted dependency genes in JHH-1 cells. The middle and right heatmaps display the average drug-to-cell attention scores in layer 1 and layer 2 for PD-184352 and YK-4279, respectively. (C) Identification of resistance-associated gene programs from attention maps. Heatmaps show representative key genes prioritized under the sensitive and resistant conditions, and the central panel lists genes enriched in the predicted drug resistance gene set. (D) Gene ontology enrichment analysis of genes preferentially prioritized in the resistant state. (E) KEGG pathway enrichment analysis of resistance-associated genes.

At the first interaction layer, the attention patterns of the two drugs were broadly similar. However, in the deeper second layer, their behaviors diverged markedly. For PD-184352, the model maintained strong attention toward genes with high VPM-predicted dependency scores, including TMEM88B, HMGCR, and DDX52 (Fig. 4B). This pattern suggests that, in the sensitive context, the model recognized and prioritized core survival vulnerabilities that are likely to mediate therapeutic efficacy. In contrast, for YK-4279, the drug-to-cell cross-attention shifted away from these essential genes and instead emphasized a distinct group of nonessential genes, including SPAG7, BRAT1, and JADE2.

To characterize the biological functions associated with this resistant state prioritization, we further examined the self-attention structure of the updated cell features after drug interaction and extracted the genes selectively emphasized in the resistant context. Approximately 20 genes, including ADAM10, BIRC2, and CAND1, were prioritized and subjected to functional enrichment analysis (Fig. 4C). Gene Ontology analysis revealed significant enrichment in pathways related to negative regulation of apoptosis and adherens junction organization, whereas KEGG analysis highlighted ErbB signaling, PD-L1 expression, and MAP kinase activity (Fig. 4D and E). Given that YK-4279 functions as an apoptosis stimulator, the prominence of antiapoptotic regulators such as BIRC2 provides a plausible mechanistic explanation for the predicted resistance phenotype. Collectively, these attention patterns highlight candidate molecular programs associated with the model’s differential predictions. Because attention weights do not by themselves establish causal relevance, these results are best viewed as hypothesis-generating signals that warrant further experimental validation.

### VCAT generalizes to independent pharmacogenomic and HCC cohorts

To assess external generalizability, we compared VCAT with four representative baselines-Random Forest, DeepCDR, PANCDR, and GCNPath-using two independent pharmacogenomic datasets and three HCC cohorts that were excluded from model training. For CTRP and PRISM, drug-cell line pairs overlapping with GDSC were removed to ensure out-of-sample evaluation. VCAT outperformed the next best models on CTRP (AUROC/AUPRC 0.759/0.451 versus 0.718/0.419) and PRISM (0.769/0.554 versus 0.726/0.493; Fig. 5A and B).

**Figure 5:**
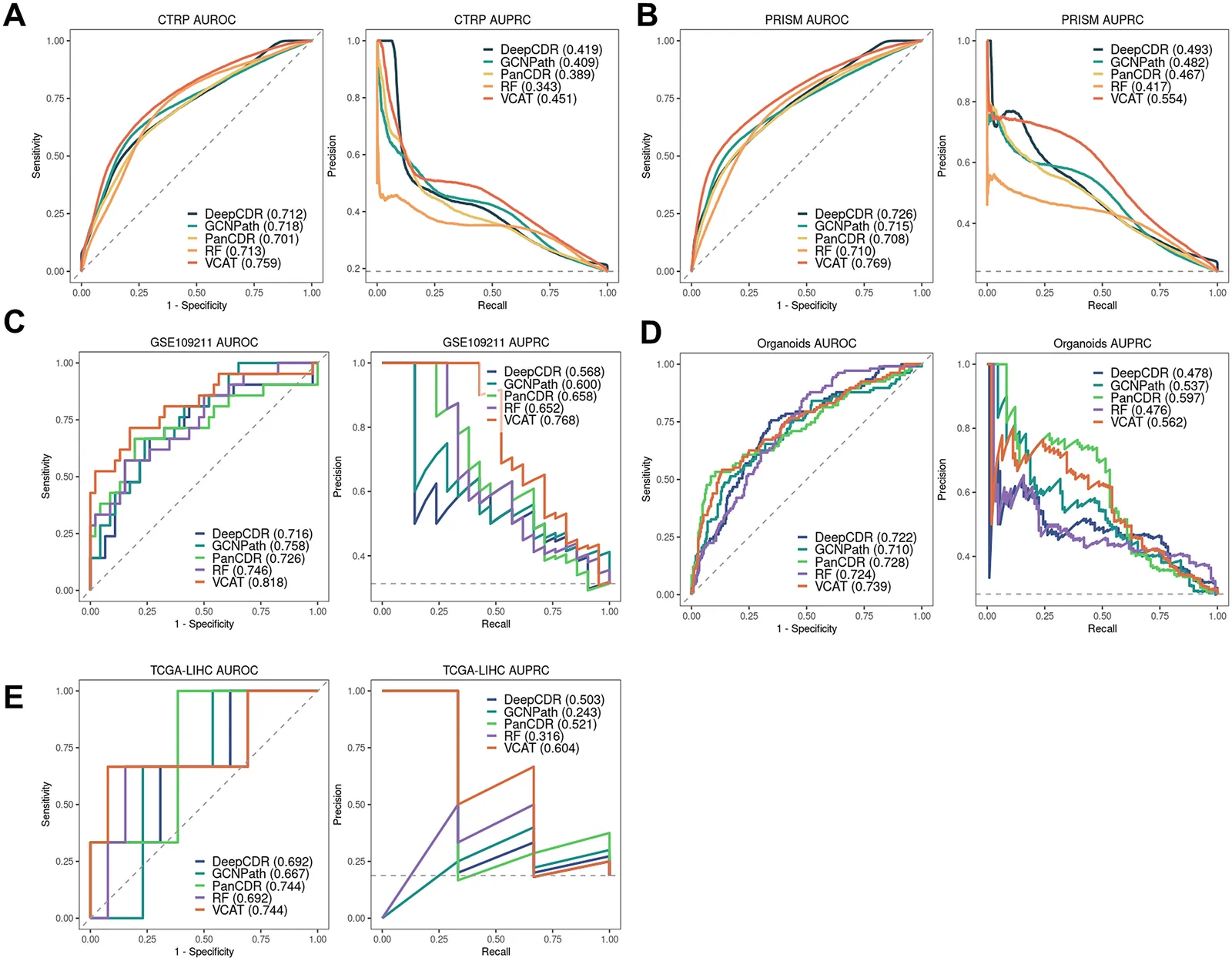
External validation of VCAT across independent pharmacogenomic and HCC datasets. Comparison of VCAT with Random Forest, DeepCDR, PANCDR, and GCNPath in (A) CTRP, (B) PRISM, (C) the GSE109211 sorafenib cohort, (D) patient-derived liver cancer organoids, and (E) TCGA–LIHC. Each panel presents the receiver operating characteristic curve (left) and precision-recall curve (right). Patient-derived transcriptomic profiles were aligned with the cell line domain using contrastive principal component analysis before prediction.

To reduce the transcriptomic domain differences between preclinical cell lines and patient derived samples, we applied linear contrastive principal component analysis before evaluating the HCC cohorts. Before alignment, the cell line and patient derived samples were clearly separated in UMAP space, whereas removal of the first four contrastive components substantially increased their distributional overlap (Supplementary Fig. S5). Genes with high loadings in the removed components were enriched in immune, cytokine response, cell adhesion, and angiogenesis related processes, indicating that these components primarily captured domain-specific microenvironmental and tissue composition signals. In GSE109211, VCAT achieved the highest AUROC and AUPRC of 0.818 and 0.768, respectively (Fig. 5C). In the liver cancer organoid dataset, VCAT achieved the highest AUROC of 0.739, although its AUPRC of 0.562 was lower than that of PANCDR (0.597; Fig. 5D). In TCGA–LIHC, VCAT achieved an AUROC of 0.744, tied with PANCDR, and the highest AUPRC of 0.604 (Fig. 5E). Overall, VCAT achieved the highest AUROC across all five external datasets, with a tie in TCGA–LIHC, and the highest AUPRC in four of the five datasets.

These findings support the generalizability of VCAT across cell line, patient derived, and organoid datasets and suggest its potential applicability to clinically relevant settings. However, given the limited cohort sizes and imbalanced response distributions in the patient datasets, particularly the small number of responders in TCGA–LIHC, further validation in larger and more balanced clinical cohorts is required.

### VCAT identifies candidate therapeutic targets associated with sorafenib resistance in HCC

Across HCC cell lines, VCAT frequently predicted sensitivity to established HCC therapies as well as additional candidate agents, while predicted responses varied substantially among individual cell lines, particularly for sorafenib (Fig. 6A and B). To investigate the molecular features associated with sorafenib resistance, we ranked genes according to their attribution scores in HCC cell lines predicted to be sorafenib resistant. Genes prioritized by the attention mechanism showed substantial concordance with those identified by IC, indicating consistency across two complementary attribution approaches (Fig. 6C and E; Supplementary Fig. S6A).

**Figure 6:**
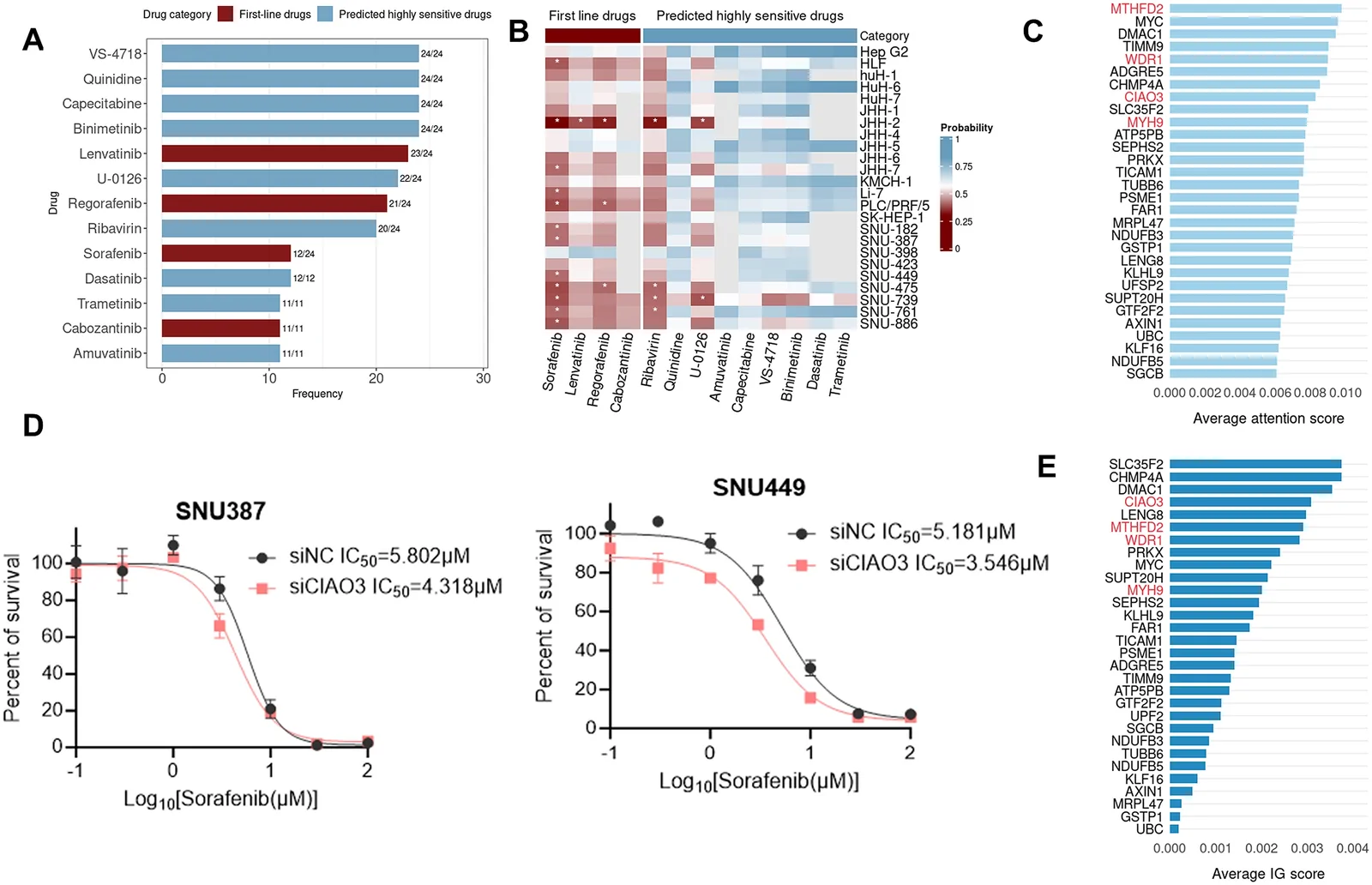
VCAT prioritizes candidate therapeutic agents and modulators of sorafenib response in HCC. (A) Frequency of drugs predicted to be highly effective across HCC cell lines. Clinically approved HCC therapies and other highly ranked candidate drugs are indicated by different colors. (B) Predicted drug sensitivity probabilities across individual HCC cell lines. Asterisks indicate predicted resistance to selected clinically approved therapies. (C) Genes ranked according to their average attention scores across predicted sorafenib-resistant HCC cell lines. (D) Sorafenib dose response curves following CIAO3 knockdown in SNU387 and SNU449 cells. (E) Genes ranked according to their average IGs scores.

Among the top ranked genes, MTHFD2 [28], WDR1 [29], and MYH9 [30] have previously been implicated in HCC progression or sorafenib response, providing biological support for the model-derived prioritization. CIAO3 was also consistently prioritized, although its involvement in sorafenib response has been less well characterized. Based on these results, CIAO3, MTHFD2, and WDR1 were selected for functional validation, whereas MYH9 was retained as a literature-supported candidate but was not experimentally tested. siRNA-mediated knockdown of each tested gene increased sorafenib sensitivity in the intrinsically resistant SNU387 and SNU449 cell lines, as reflected by leftward shifts in the dose–response curves and reductions in IC50 values (Fig. 6D, Supplementary Fig. S6B and C).

Agreement between attention and IG rankings, supported by functional perturbation, identifies biologically plausible, experimentally testable candidates associated with sorafenib response. However, attribution scores alone should not be interpreted as a evidence of causal regulatory relationships.

## Discussion

Accurate drug response prediction requires representations that capture both the biological effects of drugs and the functional state of cancer cells. In this study, we developed VCAT by integrating CTSs with CRISPR-derived vulnerability representations [15, 16]. The principal contribution of VCAT, therefore, lies in these biologically informed representations and their interaction, rather than in the use of a Transformer architecture alone. The benchmarking and ablation results demonstrate that both the input representations and the proposed interaction architecture contribute to its predictive performance.

CTS encodes downstream transcriptional effects that complement chemical structure [16, 31]. Its biological organization and representation controlled benchmarks support its value, particularly for unseen drugs, while the learned VCAT embedding remains distinct from the standardized CTS input. Vulnerability pretraining likewise contributed information beyond expression [15, 32]. Partial similarity, asymmetric cross-prediction, and improved combined-model performance indicate that the CRISPR-supervised representation reorganizes rather than duplicates baseline expression. Architectural ablations further supported adaptive fusion, cascaded interaction, and global shortcuts, although their gains complement rather than replace the biological inputs.

Attention weights were used as hypothesis-generating indicators rather than definitive causal explanations [33]. IG provided an independent attribution analysis and identified substantial agreement with the attention-based gene rankings. Functional experiments further showed that knockdown of CIAO3, MTHFD2 [28], and WDR1 [29] increased sorafenib sensitivity in hepatocellular carcinoma cell lines. MYH9 was also prioritized by the model and has previously been associated with hepatocellular carcinoma biology [30], although it was not experimentally evaluated in this study. These results strengthen the biological relevance of the prioritized genes, but validation of three genes for a single drug remains insufficient to establish the general interpretability of the framework across cancer and treatments. These findings are consistent with the broader literature describing diverse molecular and genetic mechanisms of sorafenib resistance in HCC [34, 35].

VCAT generalized across external cell line, organoid, and HCC cohorts, while continuous IC50 prediction showed that performance was not solely threshold dependent. cPCA reduced preclinical-to-patient differences [3, 17], but the small and imbalanced patient cohorts limit the strength of conclusions regarding clinical applicability. These results should therefore be considered preliminary evidence of translational potential rather than definitive clinical validation.

Several limitations should be acknowledged. CTS coverage remains incomplete, and context-specific effects may not be fully captured [36]. Although the structural overlap analysis quantified the extent of cross-split similarity, random drug identity partitioning does not ensure scaffold-level separation. The reported drug-blind performance should therefore be interpreted as generalization to held-out drug identities rather than strict extrapolation to novel chemical scaffolds. Furthermore, the balanced test sets may not reflect natural clinical response prevalence. The use of linear cPCA and the limited clinical and experimental validation are additional limitations. Future work should evaluate VCAT in larger prospective cohorts under more clinically representative settings. Further extensions could incorporate context-specific perturbations and microenvironmental information, explore foundation model and knowledge-guided representations, and model drug-combination responses [37–44].

## Conclusion

VCAT combines drug-induced transcriptional signatures with CRISPR-supervised cellular vulnerabilities for binary and continuous response prediction. Its biologically informed representations and interaction architecture provide complementary predictive gains, while external validation and functional gene perturbation support its value as a hypothesis-generating framework. Further validation in larger and more representative patient cohorts is needed to assess its clinical relevance and translational potential.

**Key Points**

- VCAT integrates LINCS derived consensus transcriptional signatures with CRISPR-informed cellular vulnerability representations for biologically grounded drug response prediction.
- Benchmarking and ablation analyses demonstrate complementary contributions from the biological representations and dual stream interaction architecture.
- VCAT supports binary and continuous IC50 prediction and generalizes across independent pharmacogenomic, organoid, and cPCA aligned HCC cohorts.
- Attention, Integrated Gradients, and siRNA experiments identify CIAO3, MTHFD2, and WDR1 as candidate modulators of sorafenib resistance.

## Supplementary Material

Supplementary_material_bbag559

## Data Availability

All datasets used in this study were obtained from publicly available resources, including DepMap, GDSC, CTRP, PRISM, CMap LINCS, TCGA-LIHC, and GEO (GSE109211). The VCAT implementation, data-processing workflows, scripts for generating consensus transcriptional signatures, and model-interpretation scripts are publicly available at https://github.com/ChrisChan99999/VCAT. Model checkpoints are available at [Zenodo 10.5281/zenodo.21637935].
