# Supplementary material for: Decoding cellular vulnerabilities for interpretable drug response prediction
Source: Brief Bioinform. 2026 Oct 15;27(5):bbag559. doi: 10.1093/bib/bbag559 (PMC13671233; doi:10.1093/bib/bbag559)
Supplement: Supplementary file 1 [file supplementary_material_bbag559.zip › Supplemental materials.docx]

**Supplemental materials**

|  | Mixed | | |  | Cell line-blind | | |  | | Drug-blind | | |  | Disjoint | | |
| --- | --- | --- | --- | --- | --- | --- | --- | --- | --- | --- | --- | --- | --- | --- | --- | --- |
| Models | AUROC | AUPRC | F1-score |  | AUROC | AUPRC | F1-score | |  | AUROC | AUPRC | F1-score |  | AUROC | AUPRC | F1-score |
| DeepCDR^†^ + CTS | 0.801 (0.012) | 0.792 (0.012) | 0.727 (0.009) |  | 0.752 (0.022) | 0.749 (0.023) | 0.678 (0.034) |  | | 0.759 (0.020) | 0.744 (0.021) | 0.686 (0.022) |  | 0.696 (0.035) | 0.696 (0.042) | 0.630 (0.032) |
|  |  |  |  |  |  |  |  |  |  |  |  |  |  |  |  |  |
| Super.FELT^†^ + CTS | 0.769 (0.009) | 0.747 (0.010) | 0.686 (0.015) |  | 0.726 (0.029) | 0.711 (0.028) | 0.659 (0.050) |  | | 0.748 (0.025) | 0.742 (0.032) | 0.661 (0.026) |  | 0.706 (0.028) | 0.710 (0.032) | 0.606 (0.046) |
|  |  |  |  |  |  |  |  |  |  |  |  |  |  |  |  |  |
| PANCDR^†^ + CTS | 0.795 (0.011) | 0.786 (0.011) | 0.720 (0.012) |  | 0.761 (0.020) | 0.754 (0.021) | 0.687 (0.035) |  | | 0.755 (0.030) | 0.746 (0.029) | 0.680 (0.049) |  | 0.704 (0.032) | 0.697 (0.042) | 0.629 (0.037) |
|  |  |  |  |  |  |  |  |  |  |  |  |  |  |  |  |  |
| DRExplainer^†^ + CTS | 0.755 (0.050) | 0.746 (0.045) | 0.688 (0.044) |  | 0.728 (0.029) | 0.714 (0.027) | 0.650 (0.047) |  | | 0.700 (0.032) | 0.696 (0.037) | 0.631 (0.047) |  | 0.691 (0.045) | 0.690 (0.042) | 0.640 (0.036) |
|  |  |  |  |  |  |  |  |  |  |  |  |  |  |  |  |  |
| GCNPath^†^ + CTS | 0.801 (0.011) | 0.791 (0.009) | 0.726 (0.010) |  | 0.741 (0.030) | 0.735 (0.032) | 0.660 (0.043) |  | | 0.763 (0.025) | 0.758 (0.028) | 0.685 (0.027) |  | 0.694 (0.028) | 0.700 (0.034) | 0.613 (0.042) |
|  |  |  |  |  |  |  |  |  |  |  |  |  |  |  |  |  |
| VCAT + SMILES | 0.802 (0.014) | 0.791 (0.011) | 0.731 (0.011) |  | 0.761 (0.020) | 0.751 (0.028) | 0.711 (0.005) |  | | 0.721 (0.035) | 0.725 (0.036) | 0.681 (0.023) |  | 0.705 (0.023) | 0.702 (0.039) | 0.673 (0.015) |
|  |  |  |  |  |  |  |  |  |  |  |  |  |  |  |  |  |

**Table S1. Performance comparison of CTS augmented deep learning models and SMILES-based VCAT across four data splitting settings.**

Values are reported as mean ± SD across ten random seeds.

**Alt text:** Representation controlled comparison of CTS augmented deep learning models and SMILES-based VCAT across four data-splitting settings.

| Split | Model | RMSE | MAE | Pearson | Spearman | R^2^ |
| --- | --- | --- | --- | --- | --- | --- |
| Cell line-blind | **VCAT** | 1.301 ± 0.034 | 0.981 ± 0.029 | 0.852 ± 0.005 | 0.814 ± 0.003 | 0.722 ± 0.014 |
| Cell line-blind | **DeepCDR** | 1.315 ± 0.010 | 0.985 ± 0.008 | 0.847 ± 0.003 | 0.807 ± 0.004 | 0.716 ± 0.006 |
| Cell line-blind | **PANCDR** | 1.324 ± 0.027 | 0.997 ± 0.016 | 0.846 ± 0.005 | 0.805 ± 0.004 | 0.712 ± 0.007 |
| Cell line-blind | **GCNPath** | 1.358 ± 0.021 | 1.022 ± 0.020 | 0.836 ± 0.005 | 0.795 ± 0.007 | 0.697 ± 0.008 |
| Cell line-blind | **Random Forest** | 1.390 ± 0.056 | 1.034 ± 0.039 | 0.827 ± 0.011 | 0.790 ± 0.012 | 0.682 ± 0.019 |
| Drug-blind | **VCAT** | 1.814 ± 0.454 | 1.349 ± 0.215 | 0.692 ± 0.170 | 0.673 ± 0.127 | 0.462 ± 0.237 |
| Drug-blind | **DeepCDR** | 2.353 ± 0.303 | 1.867 ± 0.228 | 0.472 ± 0.211 | 0.460 ± 0.148 | 0.109 ± 0.238 |
| Drug-blind | **PANCDR** | 2.410 ± 0.414 | 1.879 ± 0.367 | 0.447 ± 0.227 | 0.402 ± 0.136 | 0.061 ± 0.303 |
| Drug-blind | **GCNPath** | 2.343 ± 0.628 | 1.822 ± 0.476 | 0.453 ± 0.289 | 0.416 ± 0.255 | 0.100 ± 0.433 |
| Drug-blind | **Random Forest** | 2.193 ± 0.382 | 1.745 ± 0.276 | 0.565 ± 0.116 | 0.499 ± 0.100 | 0.247 ± 0.136 |

**Table S2. Performance comparison of VCAT and baseline models for continuous log (IC50) prediction under cell line-blind and drug-blind settings.**

Values are reported as mean ± SD across five random seeds. Lower RMSE and MAE values indicate better performance, whereas higher Pearson correlation, Spearman correlation, and R2 indicate better performance.

**Alt text:** Continuous log(IC50) prediction performance of VCAT and baseline models under cell-line-blind and drug-blind settings using five regression metrics

| Comparison | Pairs per seed | Mean Tanimoto | Median Tanimoto | ≥0.5 | ≥0.7 | ≥0.8 | Overall maximum |
| --- | --- | --- | --- | --- | --- | --- | --- |
| **Training–validation** | 2,812 | 0.121 ± 0.005 | 0.115 ± 0.005 | 0.16% | 0.04% | 0.00% | 1 |
| **Training–test** | 2,812 | 0.122 ± 0.004 | 0.117 ± 0.003 | 0.14% | 0.04% | 0.01% | 1 |
| **Validation–test** | 361 | 0.121 ± 0.007 | 0.115 ± 0.007 | 0.06% | 0% | 0% | 0.639 |

**Table S3A. Pairwise molecular similarity across the training, validation, and test drug sets.**

Pairwise molecular similarity was evaluated between drugs from the training, validation, and test sets under the drug-split setting. Molecular structures were standardized to their fragment-parent forms and represented using non-chiral ECFP4 fingerprints (Morgan fingerprints; radius = 2, 2,048 bits). Tanimoto coefficients were calculated for all drug pairs across the train-validation, train-test, and validation-test sets. The reported values summarize the similarity distributions across the 10 random split seeds. Values are presented as mean ± standard deviation across seeds where applicable.

| Held-out set | Assignments | Mean maximum Tanimoto | Median maximum Tanimoto | ≥0.5 | ≥0.7 | ≥0.8 |
| --- | --- | --- | --- | --- | --- | --- |
| **Validation** | 190 | 0.355 ± 0.021 | 0.285 ± 0.029 | 22.10% | 5.80% | 0.50% |
| **Test** | 190 | 0.350 ± 0.054 | 0.291 ± 0.035 | 20.00% | 6.30% | 1.60% |

**Table S3B. Nearest-neighbor molecular similarity and Bemis-Murcko scaffold overlap of validation and test drugs with the training set.**

For each drug in the validation or test set, the maximum Tanimoto similarity to any drug in the corresponding training set was calculated using non-chiral ECFP4 fingerprints. The table reports the distribution of these nearest-neighbor similarities, including the mean and the proportions of drugs exceeding the predefined similarity thresholds of 0.5, 0.7, and 0.8. Scaffold overlap was defined as the proportion of validation or test drugs whose Bemis-Murcko scaffold was also present in the corresponding training set. Results are summarized across the 10 random split seeds and are presented as mean ± standard deviation where applicable.

Note: The only drug pair with a non-chiral ECFP4 Tanimoto similarity of 1.0 was doxorubicin (CID 31703) and epirubicin (CID 41867). These compounds are distinct 4′-epimers but cannot be distinguished when chirality is excluded from the fingerprint calculation.

**Alt text:** Table S3A summarizes pairwise molecular similarity across training, validation, and test drug sets, and Table S3B summarizes nearest-neighbor similarity and scaffold overlap of validation and test drugs with the training set.

**Figure S1. Detailed architecture of the main modules in VCAT.** (A) Architecture of the drug adaptive encoder, including local encoding, global context encoding, feature analysis, and weighted fusion for generating drug feature embeddings. (B) Cell-conditioned self-attention layer for updating cell embeddings based on drug-conditioned information. (C) Drug-conditioned self-attention layer for updating drug embeddings based on cell-conditioned information.

**Alt text:** Detailed architecture of the adaptive CTS drug encoder and the cell-conditioned and drug-conditioned self-attention modules used in VCAT.

**Figure S2. Training dynamics of VCAT during stage 2 optimization.** Training and validation curves of VCAT during stage 2 optimization with binary cross-entropy (BCE) loss. The panels show epoch-wise changes in training loss, validation loss, combined loss comparison, AUPRC, AUROC, F1 score, accuracy, precision, and recall.

**Alt text:** Training and validation curves showing loss and classification metrics during stage-two optimization of VCAT.

**Figure S3. Seed-level performance distributions and statistical comparisons between VCAT and baseline models.** AUROC, AUPRC, and F1-score distributions are shown for the mixed, cell line-blind, drug-blind, and disjoint settings. Blue denotes comparator models and red denotes VCAT. VCAT was compared with Random Forest, DeepCDR, and GCNPath using two-sided paired Wilcoxon signed-rank tests on matched seeds, with Benjamini-Hochberg correction within each split-metric panel. *, **, and *** denote adjusted *p* < 0.05, *p* < 0.01, and *p* < 0.001, respectively.

**Alt text:** Seed-level AUROC, AUPRC, and F1-score distributions comparing VCAT with baseline models across four data-splitting settings.

**Figure S4. Redundancy and complementarity of expression and vulnerability representations.** (A) Pairwise linear centered kernel alignment (CKA) among the baseline expression, CRISPR-supervised vulnerability, fused cellular, and final VCAT representations. (B) Cross-validated R2 for predicting the vulnerability representation from expression and predicting expression from the vulnerability representation. (C) Canonical correlations between the expression and vulnerability representations across the first ten canonical components. These analyses quantify the shared and non-redundant information contained in the two cellular representations.

**Alt text:** Representation analyses showing partial similarity but substantial nonredundancy between baseline-expression and CRISPR-supervised vulnerability embeddings.

**Figure S5. Evaluation and biological interpretation of cPCA-based transcriptomic alignment.** (A) UMAP visualization of liver cancer cell lines and samples from TCGA-LIHC, GSE109211, and patient-derived liver cancer organoids before and after alignment. Alignment was performed by removing the first four contrastive principal components (cPCs). (B) Gene set enrichment analysis of the 100 genes with the highest absolute loadings in each removed cPC. Dot size represents gene-set size, color indicates −log10(FDR), and the horizontal axis shows the normalized enrichment score.

**Alt text:** UMAP plots before and after cPCA alignment and enrichment analyses of genes contributing to the four removed contrastive components.

**Figure S6. Attribution concordance and functional validation of MTHFD2 and WDR1.** (A) Rank comparison between attention and Integrated Gradients scores for the same candidate genes. The dashed line indicates identical rankings, and the experimentally evaluated genes are highlighted. (B–C) Sorafenib dose–response curves following siRNA-mediated knockdown of (B) MTHFD2 and (C) WDR1 in SNU387 and SNU449 cells. Data are presented as mean ± SEM from three independent experiments.

**Alt text:** Comparison of attention and Integrated Gradients rankings, together with sorafenib dose-response curves following MTHFD2 or WDR1 knockdown.

**Figure S7. Molecular structural overlaps across random drug-identity partitions.** (A) Distributions of pairwise molecular similarities between the training, validation, and test compound sets across ten random drug-identity splits. (B) Maximum Tanimoto similarity of each validation or test compound to its nearest structural neighbour in the corresponding training set. (C) Seed-specific proportions of held-out compounds exceeding Tanimoto similarity thresholds of 0.5, 0.7, and 0.8, together with the proportion sharing an exact Bemis-Murcko scaffold with a training compound. Molecular similarity was calculated using non-chiral ECFP4 fingerprints with a radius of 2 and 2,048 bits. Small points represent individual seeds; outlined points and error bars show mean ± SD across ten seeds.

**Alt text:** Pairwise molecular similarity, nearest-training-neighbour similarity, and scaffold overlap across ten random drug-identity splits.
